# Field experiments show no consistent reductions in soil microbial carbon in response to warming

**DOI:** 10.1038/s41467-024-45508-4

**Published:** 2024-02-27

**Authors:** Chao Yue, Jinshi Jian, Philippe Ciais, Xiaohua Ren, Juying Jiao, Shaoshan An, Yu Li, Jie Wu, Pengyi Zhang, Ben Bond-Lamberty

**Affiliations:** 1 State Key Laboratory of Soil Erosion and Dryland Farming on the Loess Plateau, Institute of Soil and Water Conservation, Northwest A & F University, Yangling, China; 2 College of Natural Resources and Environment, Northwest A & F University, Yangling, Shaanxi China; 3 College of Grassland Agriculture, Northwest A&F University, Yangling, Shaanxi China; 4 Institute of Soil and Water Conservation, Chinese Academy of Sciences and Ministry of Water Resource, Yangling, Shaanxi China; 5 Laboratoire des Sciences du Climat et de l’Environnement, LSCE/IPSL, CEA-CNRS-UVSQ, Université Paris-Saclay, Gif-sur-Yvette, France; 6 Pacific Northwest National Laboratory, Joint Global Change Research Institute at the University of Maryland–College Park, College Park, MD USA

**Keywords:** Carbon cycle, Environmental health

**arising from** G. Patoine et al. *Nature Communications* 10.1038/s41467-022-31833-z (2022)

Soil microbes play an essential role in maintaining soil functions and services, but the dynamics of soil microbial biomass carbon (MBC) under global climate change remain unclear^1^. Recently, Patoine et al.^2^ combined a global MBC data set with Random Forest modeling and reported that global MBC decreased over 1992–2013, mainly driven by increasing temperatures. Contrarily, using MBC field observations from soil warming manipulation experiments and in-situ long-term measurements across the globe, we found that MBC showed no significant changes under soil warming. Our findings indicate that soil MBC is unlikely to have decreased significantly due to the global warming of 0.28 °C during 1992–2013, and that further mechanistic studies are needed to understand potential changes in MBC under climate change.

Using a global MBC dataset^3^, Patoine and colleagues^2^ trained a Random Forest model based on spatial gradients of MBC and climate, environmental, and land-cover variables to predict the global MBC temporal change for 1992–2013. Their results showed that MBC decreased globally by 3.4% ± 3.0% (mean±95% confidence interval, with an annual decrease rate of 0.16%). Importantly, they found that temperature change is the predominant driver controlling the rates and spatial patterns of MBC change (Supplementary Figs. 1 and 5 in Patoine et al.^2^). This conclusion was based on a statistical model of largely static MBC observations, without explicitly addressing the underlying mechanisms that link warming to MBC loss.

Direct evidence for any MBC response to temperature rise should also be observed by more controlled in-situ field warming experiments. We collected 130 paired MBC measurements from such experiments via a literature survey (Supplementary Fig. 1); only two of these paired measurements were included in Patoine et al.^2^. The MBC response to warming (shown as the log-transformed response ratio, LN(RR), see Methods) exhibited no consistent decreasing trend with increased warming (Supplementary Fig. 2). Further analysis by separating the observations into different groups of warming magnitude (<1 °C, 1–2 °C, 2–3 °C, 3–4 °C, and 4–5 °C) showed that MBC only significantly decreased when soil was warmed by more than 4 °C (Fig. 1b), a level far exceeding the anthropogenic warming to date, with either no significant change or a significant increase for the warming group of 1–2 °C. We verified that these results are not affected by publication bias with the exception of the warming group of 1–2 °C (Supplementary Fig. 3; see Methods). Correcting this bias by using the “trim-and-fill” method makes the change in MBC no longer significant. The lack of a significant change in MBC in response to warming held irrespective of the warming duration (Supplementary Figs. 4 and 5).

**Fig. 1:**
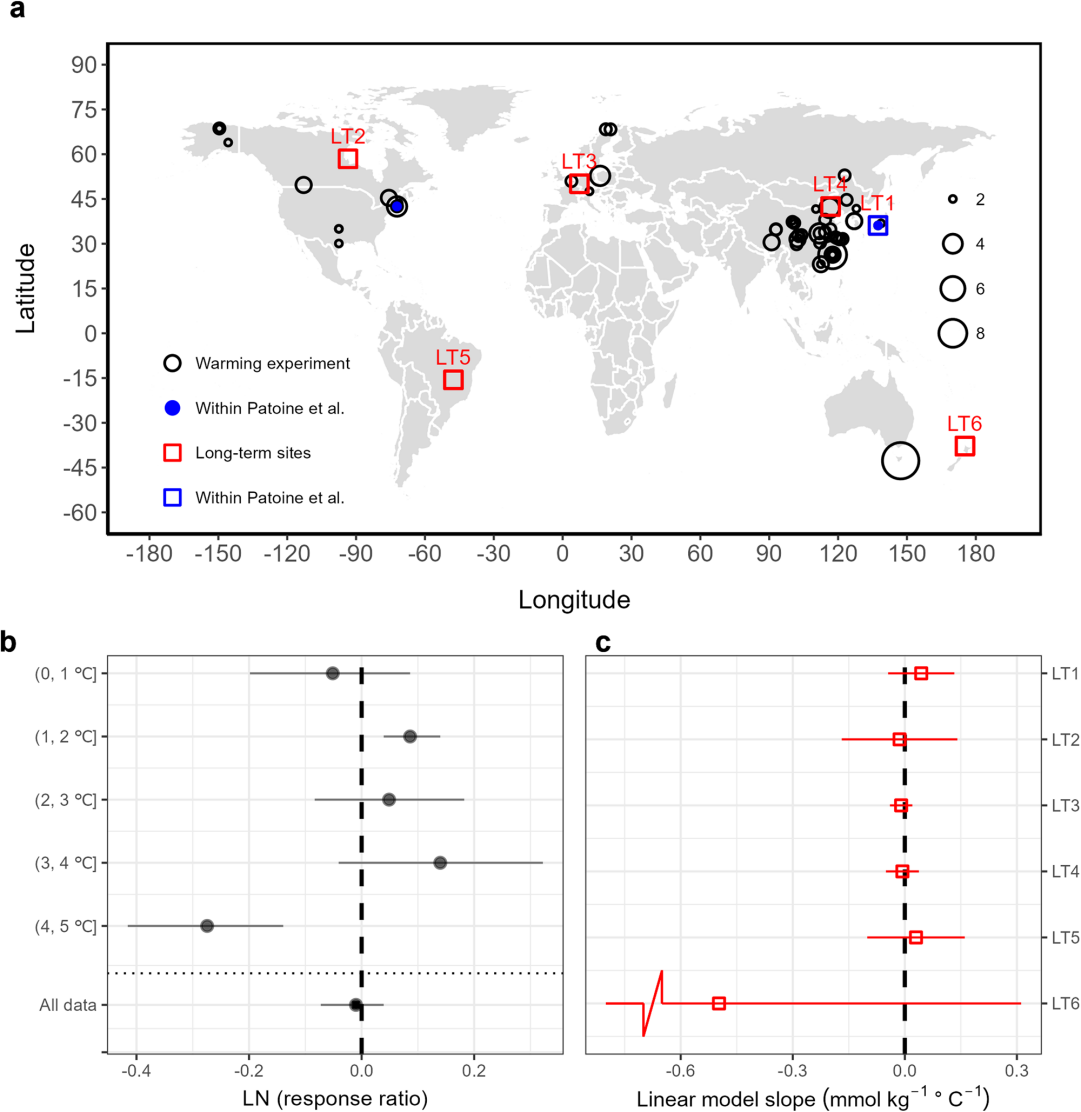
Microbial biomass carbon (MBC) changes in response to temperature change from field measurements. **a** Spatial distribution of field warming experiment sites (black circles and blue filled dots, with blue dots representing the sites included in ref. ^2^) and in-situ long-term MBC measurements (red and blue squares, with the blue square representing the site included in ref. ^2^). The circle size indicates the number of measurements. **b** MBC changes in response to soil warming (see Methods) for different bins of warming magnitude. **c** The slope of MBC against annual temperature, fitted using a simple linear regression model for each of the in-situ long-term measurement sites (Supplementary Table 1). In the panels **b** and **c**, filled dots and error bars represent the mean and the 95% confidence intervals, respectively.

The signal of MBC response to temperature change might also be detected in long-term, in-situ MBC measurements which are subject to interannual temperature changes. We tested this hypothesis by searching for in-situ long-term MBC measurements throughout the literature (Supplementary Fig. 1b). Data from six sites were collected, with the minimum observation period being 3 years but the longest period being up to 10 years (Supplementary Table 1); only one of these sites was included in Patoine et al.^2^. We found no significant correlation between MBC and annual temperature at any individual site (Fig. 1c), suggesting little change in MBC in response to temperature variation over time. This independent second line of evidence further supports the conclusion that MBC shows no significant change in response to warming.

How do we reconcile the work of Patoine et al.^2^ with these findings? The key finding of Patoine et al.^2^, of a largely continual decrease in MBC with increasing annual temperature (Supplementary Figs. 1 and 2 in Potoine et al.^2^), coincides with a negative correlation between MBC and temperature across the spatial gradient of the data used by Patoine et al.^2^ (Supplementary Fig. 6, which is also partly shown in Fig. 2a of Patoine et al.^2^). That is, the temporal decline in global MBC predicted by the Random Forest model likely emerges from the negative relationship between MBC and temperature over spatial gradients, rather than being a result of an actual change over time. Similar risks of inferring temporal sensitivities from spatial gradients (i.e., space for time substitution, SFT) have been demonstrated by Knapp et al.^4^, who argue that statistical models spatially derived from multiple sites tend to overestimate ecological responses to climatic change, compared to actual temporal models derived from in-situ multi-year observations (Fig. 1 in Knapp et al.^4^).

**Fig. 2:**
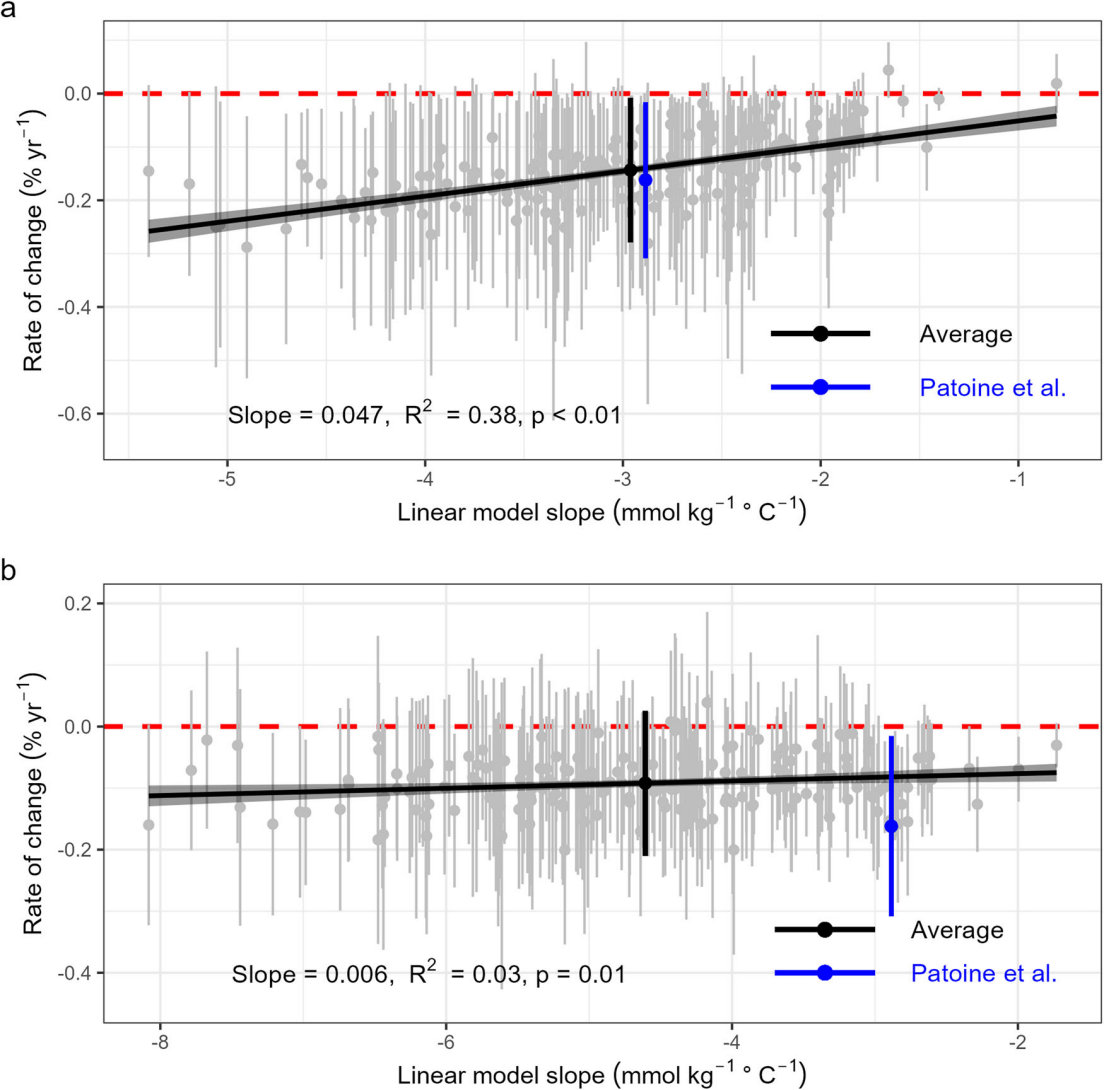
Predicted annual change rate (% yr^-1^) in global microbial biomass carbon (MBC) over 1992–2013 and its relationship with the slope between MBC and temperature. **a** Results from 200 repetitions of bootstrapping sampling (m = 500) of the original dataset (*n* = 762) of Patoine et al.^2^. Gray dots and error bars indicate the mean value and the 95% confidence interval of predicted MBC change. The average global MBC change rate from all 200 bootstrapping predictions (−0.143% ± 0.135%, mean ± 95% confidence interval) is shown by the black dot and error bar, while that reported by Patoine et al.^2^ (−0.162% ± 0.146%) is shown in blue. Panel **b** shows the same as in **a**, but in this case the 200 bootstrapping samples (m = 500) were extracted from the combined dataset (*n* = 762 + 106) of Patoine et al.^2^ and MBC observations from the control treatment of the in-situ field warming database. The average global MBC change rate from all 200 bootstrapping predictions is −0.092% ± 0.118%.

If SFT underlies the conclusion drawn by Patoine et al.^2^, then greater reductions in the global MBC are to be expected when their approach is applied to subsets of the observation data if they have steeper spatial negative slopes between MBC and temperature. We tested this hypothesis by randomly sampling from the original dataset of Patoine et al.^2^ 200 times, each time with a sample size of 500 out of the total 762 samples (for discussion of the statistical robustness of ‘m out of n’ compared to the traditional ‘n out of n’ sub-sampling in bootstrapping, please refer to Chernick^5^), followed by performing Random Forest model fitting and prediction. The change rate of global MBC during 1992–2013 determined by averaging all the bootstrapping results confirmed the reported rate in Patoine et al.^2^, but, in line with our hypothesis, the derived global MBC change rate showed significant positive correlation with the slope between MBC and temperature (Fig. 2a). Repeating the bootstrapping analysis by sampling from the combined dataset of Patoine et al.^2^ and our own data yielded a similar conclusion, but with a lower average change rate of global MBC (-0.092% ± 0.118%, Fig. 2b), only about half of that originally reported by Patoine et al.^2^.

Patoine et al.^2^ undertook an important analysis, with intuitively appealing results. Our results, however, contradict their conclusions by showing that no consistent reductions in MBC in response to warming were found in either field warming experiments or in-situ long-term measurements. Their work might potentially lead to an overestimating MBC response to a 1992–2013 average global warming of 0.28 °C. Our finding is consistent with a recent meta-analysis by Zhou et al.^6^, who found that warming does not significantly affect microbial diversity, functionality, or soil MBC, implying that microbes can adapt to certain temperature changes. On the other hand, case studies do report that long-term warming exceeding a threshold can indeed reduce substrate availability and enzyme activity, and hence reduce microbial growth and their carbon use efficiency and ultimately lead to MBC decreases^7^. Nonetheless, the large spread in LN(RR) for a given warming magnitude in our results (Fig. 1b) suggests that other factors are interacting with warming to collectively determine MBC response. We argue that collecting experimental data with more variables and integrating these with process-based models might help to improve our understanding and prediction of the fate of MBC in a warming world.

## Methods

### Methodology overview

According to Patoine et al.^2^, MBC showed a significant decreasing trend from 1992 to 2013, which was almost entirely attributed to climate change, with little contribution from land cover change. They further concluded that the climate contribution was dominated by increasing temperature rather than the change in precipitation (their Supplementary Figs. 5 and 6). This conclusion is in line with their Supplementary Fig. 1 and Supplementary Fig. 2, which show a clear decrease in MBC with increasing annual temperature, but no clear trend, or only a very slight increasing one, in MBC with increasing precipitation. Given these pieces of evidence, we decided to focus on the temperature effect on MBC in this analysis.

Here, we focus on testing three hypotheses: (1) The MBC response to warming reported by Patoine et al.^2^ should be detectable using field warming experiments, which have been widely adopted to examine how MBC responds to temperature increase. (2) Similarly, we hypothesize that the response could probably also be found in in-situ long-term MBC measurements affected by interannual temperature changes. (3) Given that the Random Forest model used to predict MBC change during 1992–2013 by Patoine et al.^2^ was trained using largely static observations of MBC stock across spatial gradients, and that a clear spatial pattern of MBC stock exists across different climatic gradients (their Fig. 2), we hypothesize that the conclusion of Patoine et al.^2^ might be subject to the space-for-time substitution (SFT) effect, in which case the predicted reduction over time could be an artifact of decreasing MBC stocks with increasing temperature over spatial gradients.

To test the initial two hypotheses, we compiled observations from field warming experiments and in-situ long-term measurements from the literature. To test the third one, we repeated the Random Forest model training followed by prediction of MBC change for 1992–2013 following the same method as Patoine et al.^2^, but used bootstrapping sub-sampling to obtain variations in both the predicted MBC change rate and the spatial slope between MBC and temperature, and further examined how the predicted MBC change rate responds to the derived spatial slope.

### Analysis using field warming experiment data

A systematic, reproducible workflow was followed to ensure the suitability and completeness of field warming experiment data included in this study (Supplementary Fig. 1a). Laboratory controlled warming experiments were excluded because they reflect the real world less realistically. Peer-reviewed articles on soil warming effects on microbial soil biomass were collected from a literature search using “soil warm” and “microbial biomass” as keywords in ScienceDirect (https://www.sciencedirect.com/), China National Knowledge Infrastructure (CNKI, https://www.cnki.net/), Google Scholar, and papers cited in previous review studies. By observing the criteria for an article to be included (Supplementary Fig. 1a), a total of 130 paired MBC measurements from both control and warming sites from 69 papers were collected (Fig. 1a).

To evaluate how MBC responds to soil warming, the effect of warming on MBC was calculated for each pair of measurements using the natural log-transformed response ratio (LN(RR)):

1 $${{{{{\rm{LN}}}}}}({{{{{\rm{RR}}}}}})={{{{\mathrm{ln}}}}}({{{{{{\rm{MBC}}}}}}}_{{{{{{\rm{t}}}}}}})-{{{{\mathrm{ln}}}}}({{{{{{\rm{MBC}}}}}}}_{{{{{{\rm{c}}}}}}})$$

Where MBC_t_ and MBC_c_ represent MBC from the warming and control treatments, respectively, and the response ratio (RR) was natural-log transformed, a common practice to make it satisfy the normal distribution^6^.

As LN(RR) seems larger for intermediate warming levels compared to either the low or high warming magnitude, potential effects of warming magnitudes on LN(RR) were examined using a quadratic fitting between LN(RR) and warming magnitude (R^2^_adj_ = 0.23, *p* < 0.01, Supplementary Fig. 2). The MBC response to soil warming was also examined in detail by separating all field-warming observations into different groups of warming magnitude (<1 °C, 1–2 °C, 2–3 °C, 3–4 °C, and 4–5 °C). The random-effect model was used to obtain the overall effect of warming on MBC and test its statistical significance (Fig. 1b). Funnel plots and the “metabias” method^8^ from the ‘meta’ package in R were employed to investigate potential publication bias for each warming magnitude group (Supplementary Fig. 3). If the funnel plot shows significant asymmetry (i.e., *p* < 0.05 derived using the “Egger” test from the “metabias” method), then an iterative “trim-and-fill” method was used to remove the most extreme publication(s) from either the left or the right tail of the funnel plot until it becomes symmetric, and then to fill imputed missing publication(s) followed by computation of a new effect size of MBC response to warming. The impacts of warming duration on MBC responses were examined similarly by grouping into different durations of <3 years, 3–6 years and 6–30 years.

### Analysis using in-situ long-term MBC measurements

We initially searched the MBC datasets used by Patoine et al.^2^ and used in a systematic analysis by Xu et al.^3^ for in-situ long-term MBC measurements, but found only one study^9^ (Supplementary Table 1 and Supplementary Fig. 1b) meeting our criteria. A subsequent systematic search in ScienceDirect, CNKI, and Google Scholar using the search terms “long-term soil microbial biomass carbon” and “soil microbial biomass carbon interannual variability” retrieved another five studies which met our criteria^10–14^ (Supplementary Table 1). For each site, annual temperatures corresponding to the observation years were retrieved from the WorldClim^15^ dataset using the recorded site location information and a linear relationship between the observed MBC and annual temperature was fitted to examine its response to changes in temperature (Fig. 1c).

### Testing the space-for-time substitution (SFT) effect in Patoine et al.^2^

According to the SFT hypothesis described above, greater predicted reductions in the global MBC are to be expected when the approach of Patoine et al.^2^ is applied to subsets of the observation data if they have steeper spatial negative slopes between MBC and temperature. Bootstrapping sub-sampling was used to verify this hypothesis: (1) 500 MBC observations were randomly taken (with replacement) from the original MBC dataset of Patoine et al.^2^ (*n* = 762) by sampling 200 times. Following the method described in Patoine et al.^2^, a Random Forest model was trained following each sub-sampling and was then used to predict global MBC for 1992–2013. For each sub-sample, the slope between MBC and annual temperature was also derived using a simple linear regression. Finally, the relationship between the predicted MBC change rate and the slope of MBC against temperature was examined. (2) similar to (1), but the dataset for sub-sampling was the dataset of Patoine et al.^2^ combined with the MBC observations from the control treatment of the field-warming dataset (*n* = 762 + 106). Only MBC observations reported in units that could be converted to mmol kg^-1^ were used, resulting in 106 measurements. The same procedure as used by Patoine et al.^2^ was then followed to derive soil MBC stocks.

In both tests, following Patoine et al.^2^, environmental variables of annual temperature, soil organic carbon, soil pH, precipitation, soil clay content, soil sand content, land-cover, soil nitrogen content, NDVI, and elevation were used as predictor variables in the Random Forest modeling. Values for these variables corresponding to the 106 control MBC measurements were extracted from the same global datasets used by Patoine et al.^2^ based on site geolocations.

To account for only those spatial grid cells where the coverage of environmental variables allows a high-confidence prediction of MBC, the spatial coverage analysis was performed for each bootstrapping sub-sampling (for both *n* = 762 and *n* = 762 + 106) following the approach of Patoine et al.^2^ (i.e., the ‘Mahalanobis distance’ approach and the ‘dissimilarity index’ approach). The results obtained by using different layers of valid pixels for model prediction for different bootstrapping sub-samplings are shown in Fig. 2. An alternative approach, using a single shared layer of valid pixels containing only collocating valid pixels of all the 200 bootstrapping sub-samplings, yielded similar results (Supplementary Fig. 7).

### Supplementary information

Supplementary Information

## Data Availability

All data used in this analysis are available at: https://github.com/jinshijian/MBC_MR.

## Code availability

The code supporting the conclusion of this study is freely available for scientific purposes and can be downloaded at https://github.com/jinshijian/MBC_MR.
