# Supplementary material for: Field experiments show no consistent reductions in soil microbial carbon in response to warming
Source: Nat Commun. 2024 Feb 27;15:1731. doi: 10.1038/s41467-024-45508-4 (PMC10899254; doi:10.1038/s41467-024-45508-4)
Supplement: Supplementary file 1 — Supplementary Information [file 41467_2024_45508_MOESM1_ESM.pdf]

Supplementary Information for:

Field experiments show no consistent reductions in soil microbial carbon in response to warming

Chao Yue[1,2], Jinshi Jian[1,3,4]*, Philippe Ciais[5], Xiaohua Ren[1], Juying Jiao[1,4]*, Shaoshan An[1,4], Yu Li[2], Jie Wu[2], Pengyi Zhang[2], Ben Bond-Lamberty[6]

[1] State Key Laboratory of Soil Erosion and Dryland Farming on the Loess Plateau, Institute of Soil and Water Conservation, Northwest A & F University, Yangling.

[2] College of Natural Resources and Environment, Northwest A & F University, Yangling, Shaanxi, China.

[3] College of Grassland Agriculture, Northwest A&F University, Yangling, Shaanxi, 712100, China

[4] Institute of Soil and Water Conservation, Chinese Academy of Sciences and Ministry of Water Resource, Yangling, Shaanxi 712100, China.

[5] Laboratoire des Sciences du Climat et de l'Environnement, LSCE/IPSL, CEA-CNRS-UVSQ, Université Paris-Saclay, 91191 Gif-sur-Yvette, France.

[6] Pacific NorthwestNational Laboratory, Joint Global Change Research Institute at the University of Maryland–College Park, 5825 University Research Court, Suite 3500, College Park, MD 20740, USA.

* Correspondence to Jinshi Jian (jinshi@vt.edu) or Juying Jiao (jyjiao@ms.iswc.ac.cn).

Supplementary Table 1. *In-situ* long-term soil microbial biomass carbon measurements used in this study.

ID	Country	Latitude (°)	Longitude (°)	Period	Included in Patoine e al. [1] or Xu et al. [2]	Reference
LT1	Japan	36.13	137.42	2010-2011, 2013-2015	Yes	Suzuki et al. [3]
LT2	Canada	58.44	-93.48	2004-2007	No	Edwards and Jefferies [4]
LT3	Germany	50.12	7.31	1986-1995	No	Emmerling et al. [5]
LT4	China	42.45	116.67	2005-2007	No	Liu et al. [6]
LT5	Brazil	-15.60	-47.60	2011, 2013, 2015	No	Lopes et al. [7]
LT6	New Zealand	-37.80	175.25	1990-1997	No	Wardle et al. [8]

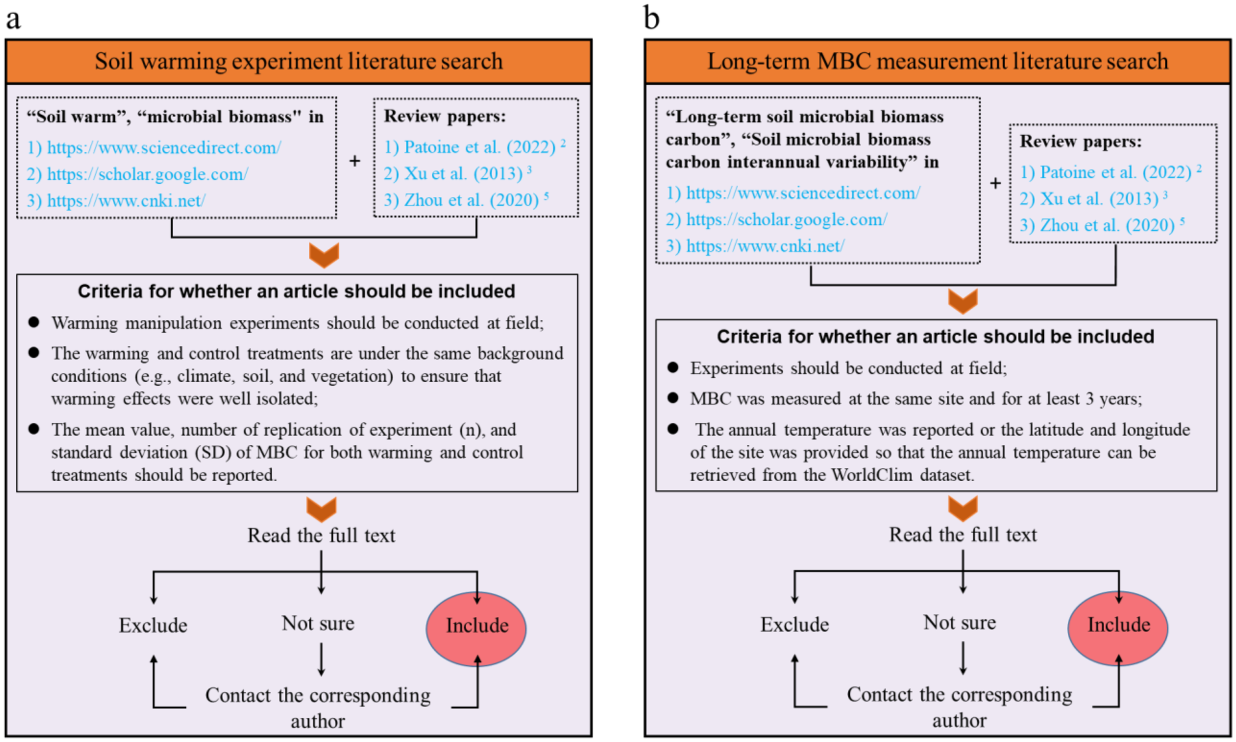

Supplementary Figure 1. The literature searches and data collection workflow.

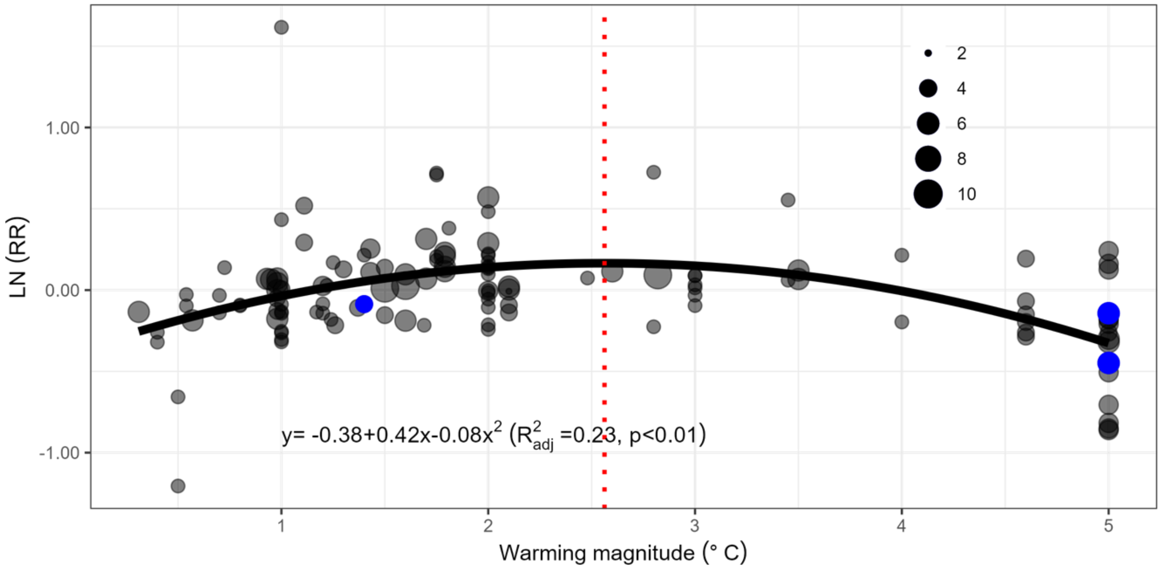

Supplementary Figure 2. The relationship between the natural log-transformed microbial biomass carbon response ratio (LN(RR)) derived from field warming experiments and the magnitude of warming, fitted with a quadratic curve. Blue dots represent sites included in Patoine et al. [1]; black dots represent data collected in this study. The dot size indicates the number of measurements, with the blue dots representing the site included in ref [1].

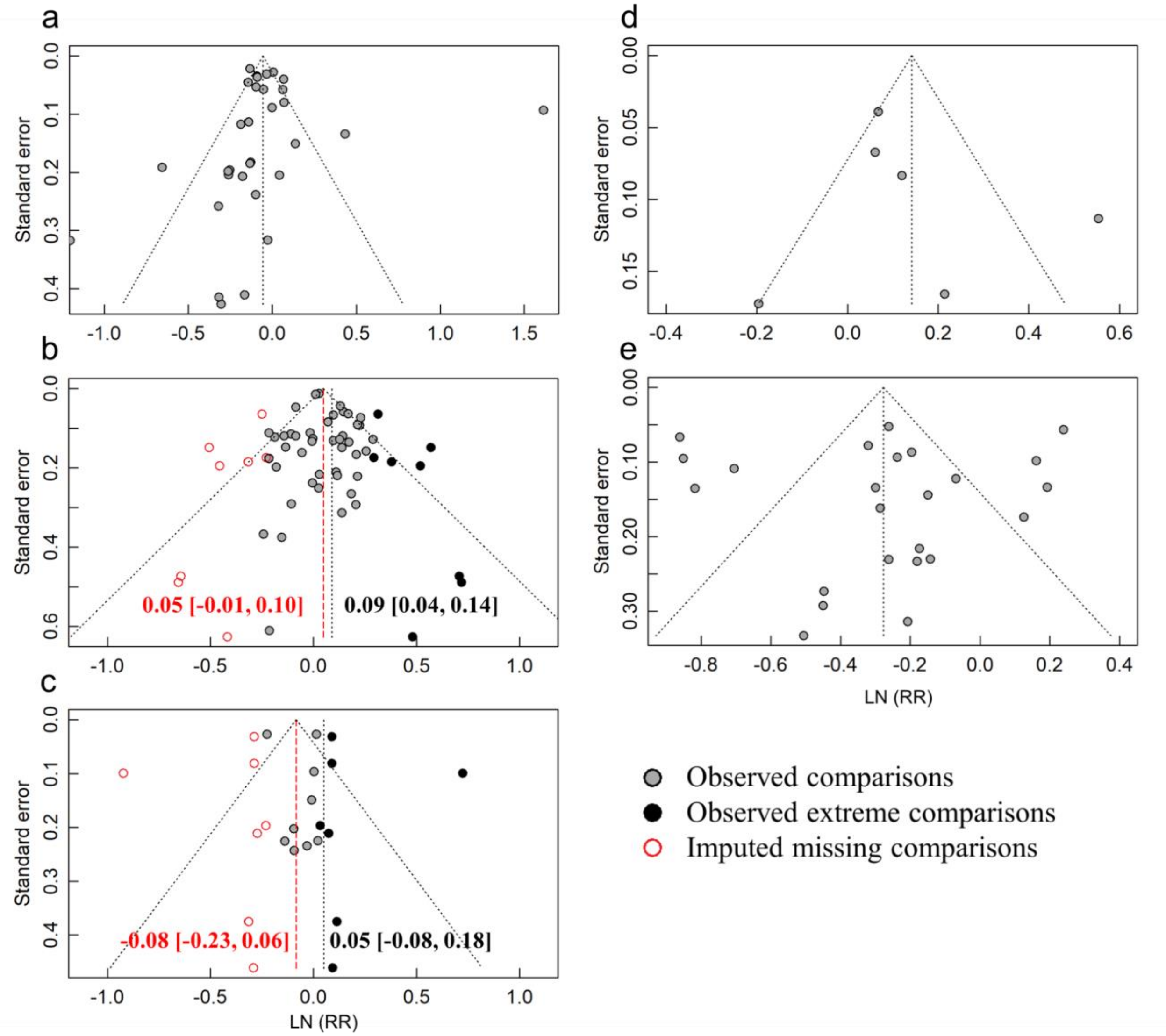

Supplementary Figure 3. Potential publication bias in the response of soil microbial biomass carbon (MBC) to field warming (natural log-transformed response ratio, when assessed using the "trim-and-fill" method for different warming magnitudes. (**a**)-(**e**) are the funnel plots for warming magnitudes of (0,1], (1,2], (2,3], (3,4], and (4, 5] °C, respectively. The grey solid dots are the observed comparisons, the black solid dots are the observed extreme comparisons detected by the "trim-and-fill" method, the red open dots are the missing comparisons imputed by the "trim-and-fill" method. The numbers in black and red are the mean value and 95% confidence intervals before and after applying the "trim-and-fill" method, respectively.

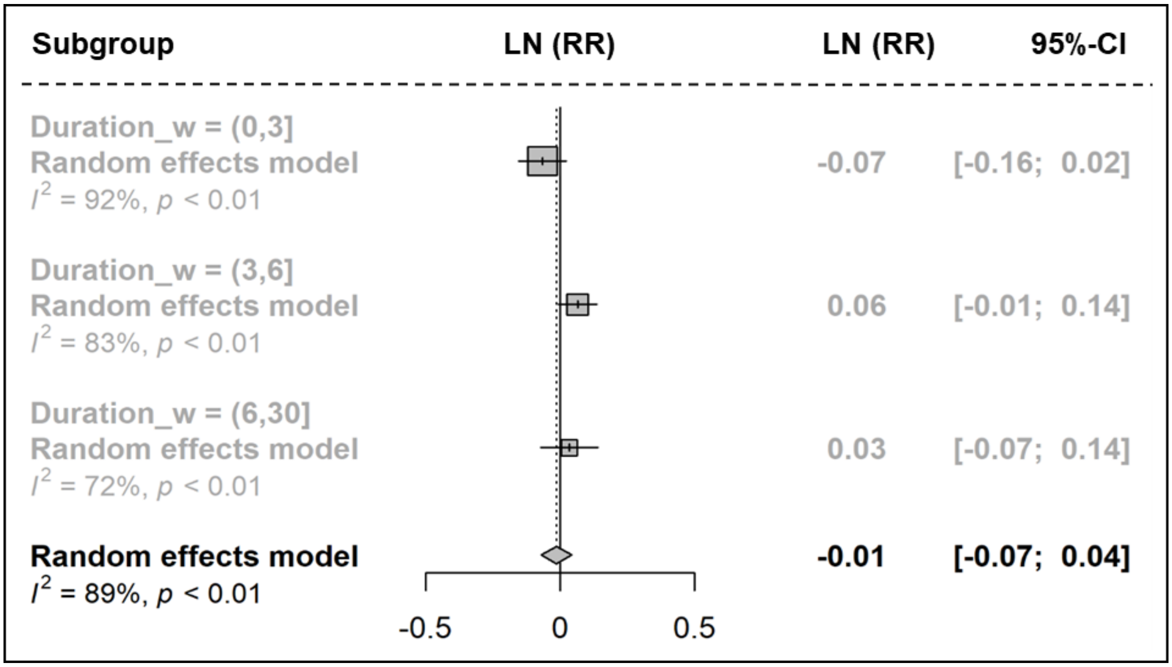

Supplementary Figure 4. Effects of warming duration on the response of soil microbial biomass carbon (MBC) to warming (natural log-transformed response ratio, LN(RR)). Data were grouped into different field warming durations of less than 3 years (0,3], between 3 and 6 years (3,6], and longer than 6 years (6,30].

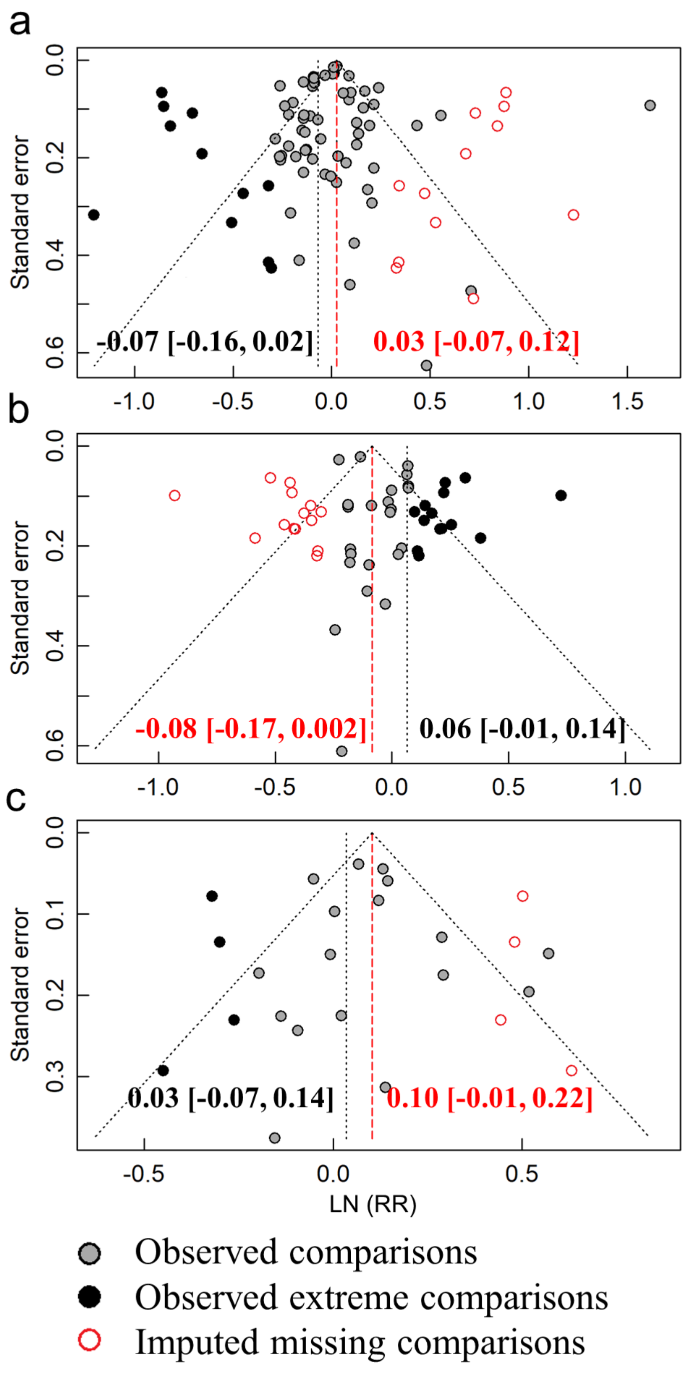

Supplementary Figure 5. Potential publication bias in the response of soil microbial biomass carbon (MBC) to field warming (natural log-transformed response ratio, LN(RR)) assessed using the "trim-and-fill" method for different warming durations. (**a**)–(**c**) are the funnel plots for warming durations of (0,3], (3,6] and (6,30] years, respectively. The grey solid dots are the observed comparisons, the black solid dots are the observed extreme comparisons detected by the "trim-and-fill" method, and the red open dots are the missing comparisons imputed by the "trim-and-fill" method. The numbers in black and red are the mean value and 95% confidence intervals before and after applying the "trim-and-fill" method, respectively.

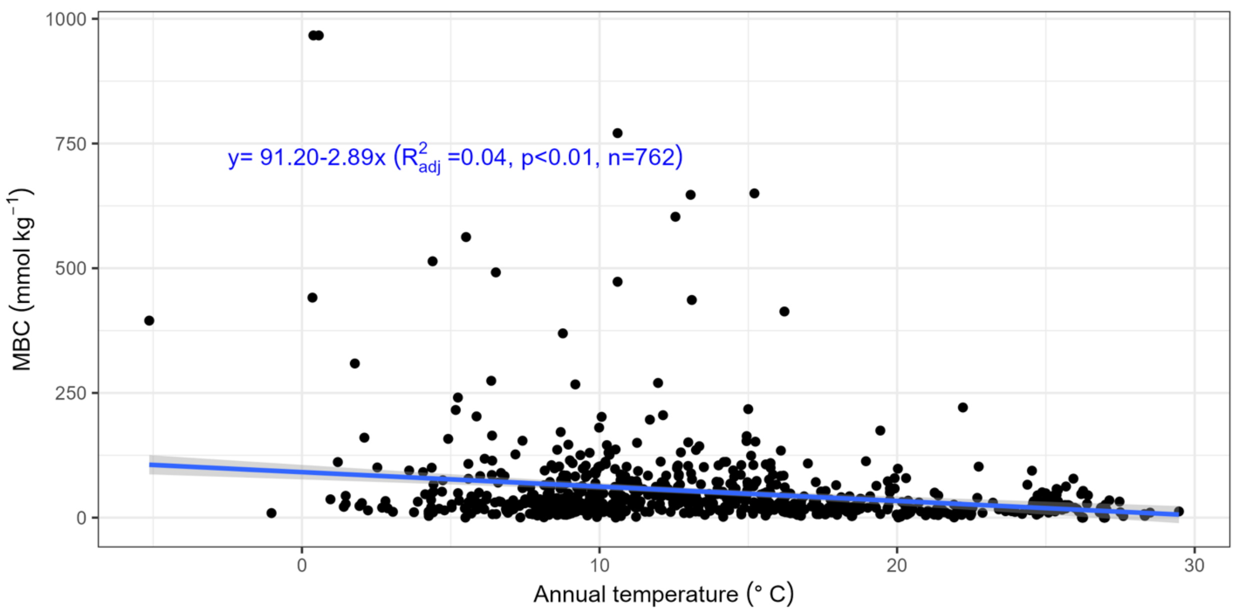

Supplementary Figure 6. Relationship between soil microbial biomass carbon (MBC) and annual temperature based on the data from Patoine et al.[1].

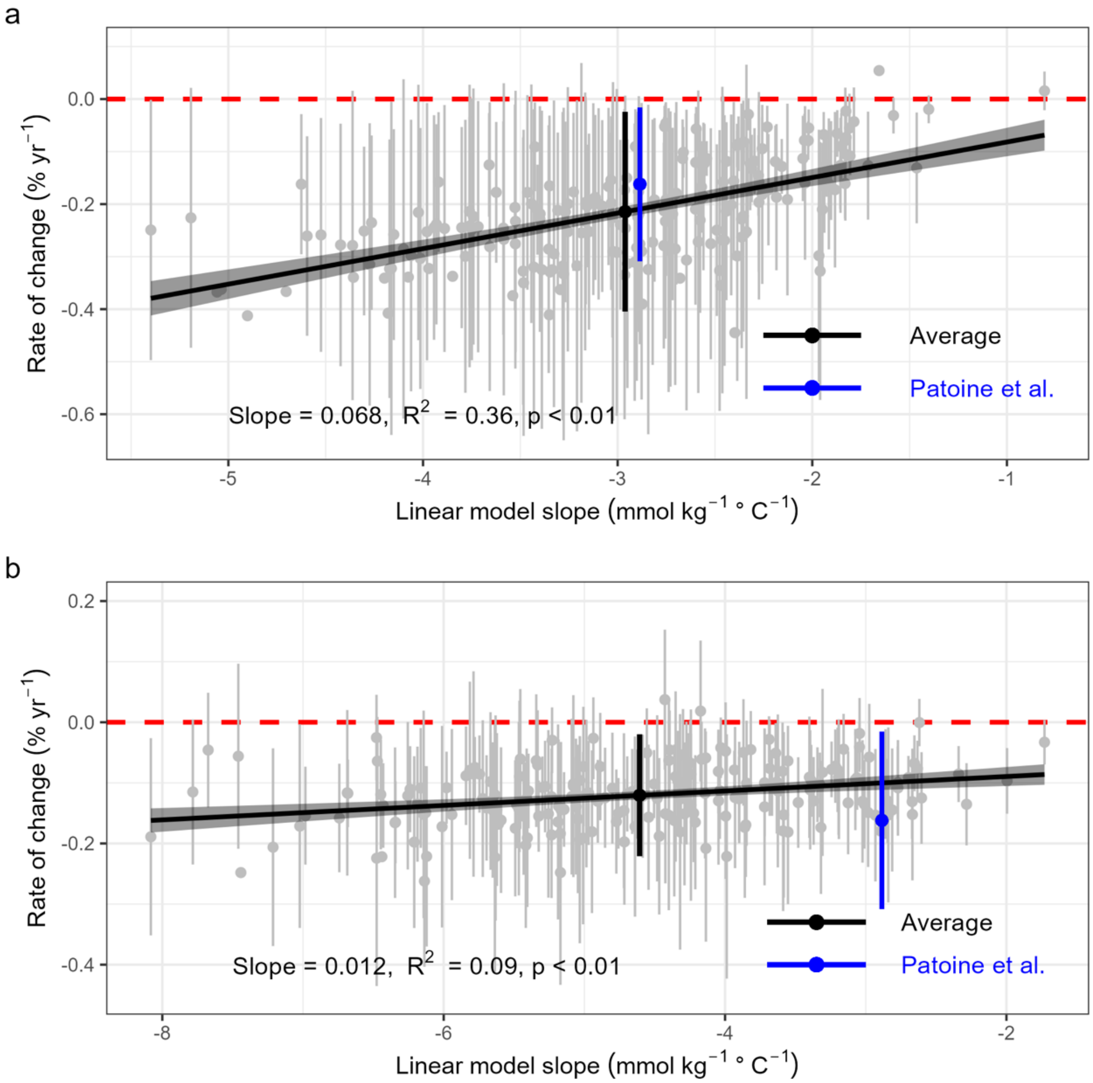

Supplementary Figure 7. Predicted annual rates of change (% yr^{-1}) in global microbial biomass carbon (MBC) over the period 1992–2013 and their relationships with the slope between MBC and temperature. Same as Figure 2 in the main text, but all 200 bootstrapped predictions of global MBC used a single shared layer of valid pixels which contains only collocating valid pixels of all the 200 bootstrapping sub-samplings.

References

1. Patoine, G. *et al.* Drivers and trends of global soil microbial carbon over two decades. *Nat. Commun.* **13**, 4195 (2022).
2. Xu, X., Thornton, P. E. & Post, W. M. A global analysis of soil microbial biomass carbon, nitrogen and phosphorus in terrestrial ecosystems. *Glob. Ecol. Biogeogr.* **22**, 737–749 (2013).
3. Suzuki, M. *et al.* Effects of long-term experimental warming on plants and soil microbes in a cool temperate semi-natural grassland in Japan. *Ecol. Res.* **31**, 957–962 (2016).
4. Edwards, K. A. & Jefferies, R. L. Inter-annual and seasonal dynamics of soil microbial biomass and nutrients in wet and dry low-Arctic sedge meadows. *Soil Biol. Biochem.* **57**, 83–90 (2013).
5. Emmerling, C., Udelhoven, T. & Schröder, D. Response of soil microbial biomass and activity to agricultural de-intensification over a 10 year period. *Soil Biol. Biochem.* **33**, 2105–2114 (2001).
6. Liu, W., Xu, W., Hong, J. & Wan, S. Interannual variability of soil microbial biomass and respiration in responses to topography, annual burning and N addition in a semiarid temperate steppe. *Geoderma* **158**, 259–267 (2010).
7. Castro Lopes, A. A. *et al.* Temporal variation and critical limits of microbial indicators in oxisols in the Cerrado, Brazil. *Geoderma Regional* **12**, 72–82 (2018).
8. Wardle, D. A., Yeates, G. W., Nicholson, K. S., Bonner, K. I. & Watson, R. N. Response of soil microbial biomass dynamics, activity and plant litter decomposition to agricultural intensification over a seven-year period. *Soil Biol. Biochem.* **31**, 1707–1720 (1999).
